# Investigating the Relationship Between Creativity and Entrepreneurial Intention: The Moderating Role of Creativity in the Theory of Planned Behavior

**DOI:** 10.3389/fpsyg.2020.01209

**Published:** 2020-06-09

**Authors:** Yongchuan Shi, Tulin Yuan, Robin Bell, Jiatong Wang

**Affiliations:** ^1^College of Innovation and Entrepreneurship, Wenzhou University, Wenzhou, China; ^2^College of Education, Wenzhou University, Wenzhou, China; ^3^Worcester Business School, University of Worcester, Worcester, United Kingdom; ^4^Department of Technology Integration and Entrepreneurship, Kunsan National University, Kunsan, South Korea

**Keywords:** creativity, entrepreneurial intention, TPB model, university students, entrepreneurship education

## Abstract

Cultivating students’ creativity in entrepreneurship education at the college and university level is a key facet of entrepreneurship education in encouraging innovation in students. In this study, the influence of creativity, self-efficacy, entrepreneurial attitude, perceived control, and subjective norms, on students’ entrepreneurial intention were examined through a moderated model based on Ajzen’s (1985) Theory of Planned Behavior (TPB). A questionnaire survey was used to obtain the data from 523 students from different universities in China’s Zhejiang province. SPSS 20.0 was used to conduct descriptive analysis and exploratory analysis of the data, and Amos 22.0 was used to conduct confirmatory factor analysis. The research concluded that creativity has a significant impact on entrepreneurial intention; entrepreneurial self-efficacy has a marked effect on perceived behavior control; and perceived behavioral control, subjective norms and entrepreneurial attitude all significantly affect entrepreneurial intention. Finally, creativity has a significant moderating effect on the roles of perceived behavioral control and subjective norms on entrepreneurial intention, but not on the attitude to entrepreneurship. These results suggest that entrepreneurship education should focus on the cultivation of students’ creativity and entrepreneurial efficacy, while encouraging their entrepreneurial intention as well as developing their entrepreneurial skills and mindset.

## Introduction

As a conscious, planned, risky, and complex decision-making behavior, entrepreneurship is influenced by many factors in the process, among which creativity is a primary condition and entrepreneurial intention is an important driving factor for entrepreneurial behavior and action.

Intention is an essential prerequisite for individuals’ actions. Not all potential entrepreneurs will start their own businesses after spotting opportunities; entrepreneurship is driven by entrepreneurial intention. The greater the individual’s intention to undertake a given behavior then the more likely it will be effectively executed (Maresch et al., 2016). Moreover, entrepreneurship is a path-breaking value creation process where entrepreneurs are characterized by innovative intellect. In Schumpeter’s (1942) innovation theory, the essence of “creative destruction” lies in the creativity of entrepreneurs. Creativity, from the perspective of entrepreneurship, is reflected in the process of developing original and practical ideas to create new enterprises, or projects, and then bringing about new products or services (Mumford, 2003). As entrepreneurial intention serves as the prerequisite or critical step for entrepreneurship (Zhao et al., 2010), is it possible for creativity to inspire individuals’ entrepreneurial intention?

The paper is structured as follows. The section entitled, “Literature Review and Hypothesis Development” briefly reviews previous studies that touch on the relationship between creativity and entrepreneurial intention. The following section, “Aims and Hypotheses” introduces our hypotheses. The methodology and data are then presented in the section, “Materials and Methods.” The “Results” section then presents the relationships among the targeted variables. The paper concludes with a discussion of the contributions and limitations of this study in the final “Discussion and Conclusion” section.

## Literature Review and Hypothesis Development

### The Theory of Planned Behavior Model

The Theory of Planned Behavior (TPB) was proposed by Ajzen (1985) through his article *From Intentions to Actions: A Theory of Planned Behavior*. According to the TPB Model, there are three attitude variables that affect entrepreneurial intention, which are the attitude toward the behavior, subjective norms and perceived behavioral control. This theory is developed from the theory of rational action. Since it was put forward, it has been widely used in the research of belief, attitude, behavior intention and other fields, having a significant impact particularly on consumption, public relations, health care, career choice and other predictions of social behavior. In recent years, due to the rise of entrepreneurship research in the world, the TPB has been rapidly applied to the research of entrepreneurship. Based on the TPB, entrepreneurial behavior can be explained as follows: The level of entrepreneurial intention is related to the attitude to the behavioral intention of entrepreneurs (attitude toward the behavior); the level of entrepreneurial intention is related to normative belief and compliance motivation (subjective norms); the level of entrepreneurial intention is related to control belief and perceived facilitating conditions (behavioral control). Liñán (2004) described the three entrepreneurial intent attitude variables in terms of personal preference or attractiveness of the idea; perceived social norms; and perceived entrepreneurial effectiveness. When these conditions are sufficient, the entrepreneurial intention of entrepreneurs is at a higher level, thus entrepreneurs are more likely to start their business. Since attitudes can change over time entrepreneurial intent can change as the individual’s perceptions change. Such changes can occur, for example, through education or experience which have the potential to increase self-efficacy and perceived entrepreneurial effectiveness, and desirability (Liñán et al., 2011; Bell and Bell, 2016). Perceived subjective norms can be considered as the individual’s perception of the opinion of other people (important to the individual) on the behavior. Aldrich and Cliff (2003) opined that the characteristics of the family system, including norms, values and family resources could impact new venture creation and Edelman et al. (2016) that social capital together with emotional social support can markedly affect entrepreneurial engagement and progression. Positive support and social norms (social pressures) can thus also encourage intent, whilst negative ones may discourage it.

Some scholars realized that environment or individual level variables could not adequately explain entrepreneurial behavior. They used the TPB model and entrepreneurial event model to discuss the key factors that affect entrepreneurial intention. In order to verify the effectiveness of the three attitude variables, they also introduced three pre-variables that were expected value, normative belief and self-efficacy (Krueger et al., 2000). At the same time, many studies had confirmed that the TPB could explain entrepreneurial intention (Kautonen et al., 2013). Based on the TPB, Moriano et al. (2012) conducted research on entrepreneurial intention across various cultural backgrounds. They selected 1,067 students from Germany, India, Iran, Poland, Spain, and Netherlands as samples to compare. The results supported the influence of attitude and perceived behavior control (self-efficacy) on entrepreneurial intention and the influence of cultural differences on subjective norms. Yang (2013) applied TPB to survey the entrepreneurial intention of 1,330 Chinese students. The results showed that attitude is the most effective predictor of entrepreneurial intention, followed by subjective norms, and then perceived behavioral control. Maes et al. (2014) conducted a survey on business school students’ entrepreneurial intention by using TPB and building a structural equation model. The results showed that the influence of gender on entrepreneurial intention was regulated by personal attitude and perceived behavioral control, but not by social norms. The ideal stage of learning and cultivating a positive attitude toward entrepreneurship should be in childhood and adolescence. However, most of the research subjects are college students rather than middle school students. Based on the revised plan behavior theory, Xu et al. (2016) adopted a stratified cluster sampling method to conduct a survey of entrepreneurship education in 1,018 middle schools in China, to investigate the impact of entrepreneurship education on the attitude, subjective norms, perceived behavioral control and entrepreneurial intention of middle school students. In order to confirm the determinants of academic entrepreneurial intention, Feola et al. (2019) adopted the structural equation model and triple helix model to test the model with Italian researchers. The results emphasized that all psychological variables of TPB were related to the prediction of academic entrepreneurial intention. The research results of Dai and Chen (2017) showed that college students’ entrepreneurial perception of behavior control had a significant impact on entrepreneurial behavioral intention.

### Creativity

Creativity is an important component of individual cognitive processing, and has the ability to generate new and valuable ideas by recombining and matching information and knowledge (Zhang and Zhang, 2018). The divergence in the academic circle on the definition of creativity is wide. There are more than 100 definitions of creativity in various literatures (Meusburger, 2009). Creativity theory defined the leading factors affecting creativity as “four P’s,” namely process, product, person and place (Rhodes, 1961). In the cognitive method, researchers paid special attention to the process of creativity and tried to describe the mechanism and technology of creative thinking. For example, Torrance (1966) defined creativity as a process, that is, first, a person is aware of problems, defects, and disagreements which are difficult to identify, and then it is necessary to find solutions and put forward hypotheses, and finally to test and modify these hypotheses to deliver a successful outcome. With the emergence of the psychological measurement method of creativity initiated by Guilford et al. (1952), many studies also showed that creativity actually involved more abilities, such as the ability of openness, hierarchical thinking, autonomy, exploratory behavior, etc. If environmental factors are taken into account, creativity will be associated with factors such as autonomy and resource access. The characteristics of a creative lifestyle are unqualified attitude, behavior, and flexibility (Sternberg et al., 2012).

In the field of economic production, many scholars pay more attention to the relationship between creativity and products. For example, Mumford (2003) thought that creativity involved the production of novel and useful products, while Sternberg et al. (2012) suggested that creativity meant the production of “original and valuable things.” Some economists regard creativity as an important element of recombining elements to generate new technologies and products, to promote economic growth (Bloom et al., 2013). Therefore, the impact of creativity on the economy should not be ignored.

With the emergence of entrepreneurship, many scholars associate creativity with entrepreneurship because creativity is particularly crucial for entrepreneurial activities, and entrepreneurship itself is a creative activity. Remaining creative is a quality that a successful entrepreneur must have. In the field of entrepreneurship, creativity at an individual level refers to the process in which entrepreneurs can combine existing resources and generate new ideas to start innovative businesses (Chua and Bedford, 2016). Scholars are used to studying the relationship between creativity and entrepreneurship in the framework of organizational management. Social psychologists, organizational scientists, and management scientists have conducted extensive research on the relevant factors affecting the creativity of teams and organizations, and developed various comprehensive theoretical models, and emphasized the role of team composition, team process and organizational culture, and their interaction with promoting innovation (Woodman et al., 1993; Paulus and Dzindolet, 2008; Salazar et al., 2012; Harvey, 2014).

### Entrepreneurial Intention

The study of entrepreneurial intention is a rapidly developing area of research (Liñán and Fayolle, 2015) and research suggests that entrepreneurial intention is an important precursor in becoming an entrepreneur (Zhao et al., 2010). Intention is a key antecedent of action, and the study of entrepreneurial intention can deepen people’s understanding of entrepreneurial cognition and behavior patterns. The formation of entrepreneurial intention is the product of the interaction between individuals and the environment, and its relative research focuses more on the influencing factors of entrepreneurial intention (Sun et al., 2011). Starting from personal characteristics or external environment, researchers explored various factors that may lead to entrepreneurial intention, and studied the influencing mechanism. Some scholars apply the decision-making model to the study of entrepreneurial intention. For example, Simon et al. (2000) investigated whether some individuals were engaged in entrepreneurship because their cognitive biases (mental short cuts) led them to perceive lower risks than might be the case. Based on a study of 192 students they opined that individuals often start ventures because they do not perceive the risks (rather than knowingly accept high levels) because cognitive biases such as a belief in the ‘law of small numbers’ (limited information) or an inflated illusion of control reduced their perception of risk.

In addition, some scholars have studied entrepreneurial intention based on social cognitive theory. Zhao et al. (2005) took self-efficacy as a key antecedent to influence entrepreneurial intention, and explored how factors such as formal learning perception, entrepreneurial experience, risk preference and gender, affected the formation of entrepreneurial intention by influencing entrepreneurial self-efficacy. They collected data from two rounds of MBA student samples. The empirical results illustrated that formal learning perception, entrepreneurial experience and risk preference can enhance entrepreneurial intention by improving entrepreneurial self-efficacy, while the effect mechanism of gender on entrepreneurial intention was relatively complex. Although gender differences do not bring differences in individual entrepreneurial self-efficacy, it can directly affect entrepreneurial intention, that is, women’s entrepreneurial intention was lower than men’s. The excessive attention to individual factors has led some scholars to fail to explore the environment as a factor affecting entrepreneurial intention. Therefore, with the continuous expansion of the research field, some scholars have begun to study the impact of environmental factors. In many studies, scholars have used the TPB to investigate the environmental factors.

### Creativity and Entrepreneurial Intention

In recent years, many scholars have begun to pay attention to the influence of creativity on entrepreneurial intention. Being entrepreneurial enables entrepreneurs to better understand the connection between things, identify business opportunities and the rational allocation of entrepreneurial resources, so as to smooth the path of value creation. People with high creativity can maintain a positive attitude and high self-confidence in entrepreneurial activities. As creativity involves individual traits and abilities, many scholars also combine creativity to study entrepreneurs’ intention to start their own businesses. Hamidi et al. (2008) introduced creativity into the theoretical model of entrepreneurship education and entrepreneurial intention for the first time, using multiple and ordered regression analysis to test the hypotheses derived from the theory. The results showed that creativity exercises could improve students’ entrepreneurial intentions. Zampetakis et al. (2011) studied the connection between young people’s creativity and entrepreneurial intentions in a survey of 180 undergraduate business school students, and found that the more creative young people thought they were, the higher their entrepreneurial intentions were. Chia and Liang (2016) conducted a survey of the impact of creativity on entrepreneurial intention at a university in Taiwan, which divided the creativity of tourism students into two dimensions, namely, originality and practicality, and showed that students with higher creativity mirrored greater entrepreneurial intention. Miranda et al. (2017) used TPB to study the impact of attitudes, subjective norms and perception control on scholars’ entrepreneurship intentions. They conducted a survey of 1,178 Spanish university scholars from different institutions, professions and qualifications, and found that entrepreneurial intention was influenced by creativity, perceptual utility and entrepreneurial experience, and that creativity can have a positive impact on entrepreneurial attitudes. Hu et al. (2018) explored the extent to which entrepreneurial alertness regulated the impact of students’ proactive personality and creativity on entrepreneurial intentions. Through field surveys of 735 undergraduates at 26 Chinese universities, they demonstrated that entrepreneurial alertness had an absolute mediating effect between creativity, proactive personality and entrepreneurial intention. According to the TPB and entrepreneurship event models, Zhao et al. (2005) explained the logic of the impact of creativity on entrepreneurial intentions as, people with high creativity could maintain a positive attitude and high self-confidence in entrepreneurial activities. In today’s environment of encouraging entrepreneurship, intangible social norms will also support people to choose creative work. Despite creativity being highlighted as an important resources for entrepreneurs (Ahlin et al., 2014; Khedhaouria et al., 2015), research has yet to fully explore the role of creativity in the TBP. This research is designed to fill this gap and investigate the impact of creativity on entrepreneurial intention, and the moderating role of creativity, through the theory of TPB.

## Aims and Hypotheses

**FIGURE 1:**
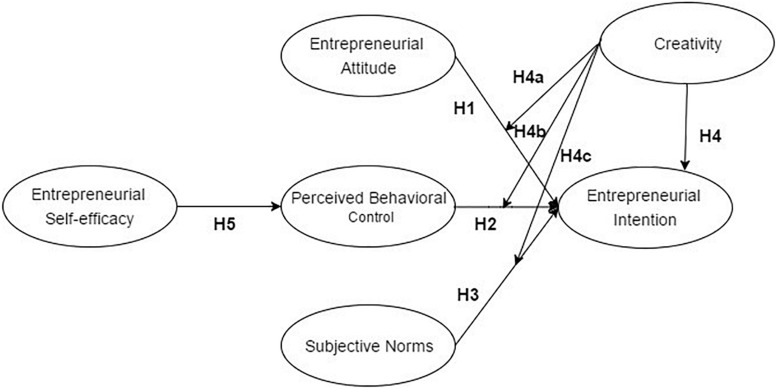
The proposed structural relationships between creativity and entrepreneurial intention based on the theory of planned behavior model.

As discussed in the previous sections, the relationships between creativity and entrepreneurial intention remain under-explored. Therefore, this study is based on the moderated TPB model, which has been extensively applied in the study of entrepreneurial intention, and has a good ability to explain the factors that influence entrepreneurial intention. The theoretical model of this study takes self-efficacy as the pre-variable of perceived behavioral control. It also introduces the variable of creativity to explore the influence of creativity on entrepreneurial intention, and determine whether creativity has a moderating effect on the relationship between entrepreneurial attitude, perceived behavior control, and subjective norms, on entrepreneurial intention (Figure 1). Based on this theoretical model, a questionnaire survey was conducted, and the relationship between creativity and entrepreneurial intention has been further explored. The hypotheses in this study are as follows:

H1: Entrepreneurial attitude has a positive effect on entrepreneurial intention.
H2: Perceived behavior control has a positive effect on entrepreneurial intention.
H3: Subjective norms have a positive effect on entrepreneurial intention.
H4: Creativity has a positive effect on entrepreneurial intention.
H4a: Creativity plays a positive role in moderating entrepreneurial attitude and entrepreneurial intention.
H4b: Creativity plays a positive role in moderating perceived behavior control and entrepreneurial intention.
H4c: Creativity plays a positive role in moderating subjective norms and entrepreneurial intention.
H5: Entrepreneurial self-efficacy has a positive effect on perceived behavior control.

## Materials and Methods

### Participants

This study, through SPSS20.0, carried out a descriptive analysis of the respondents’ gender, school, grade, profession, and whether there were entrepreneurs in their family etc. Of the undergraduate students surveyed, 43.79% were male and 56.21% were female. In terms of grades, freshmen students accounted for 54.68%, sophomores 24.67%, juniors 13%, and seniors 7.65%. In terms of major distribution, economic management accounted for 49.08%, science and engineering accounted for 25.62%, sports accounted for 0.4%, art accounted for 4%, philosophy and sociology accounted for 1.34%, literature and history accounted for 6.69%, and medicine and surgery accounted for 15.87%. In terms of entrepreneurs in the family, those with entrepreneurs accounted for 37.48%, and those without entrepreneurs accounted for 62.52%.

### Instruments

This study was based on the measurement of 6 latent variables via 48 questions: entrepreneurial intention (four questions), entrepreneurial attitude (four questions), perceived behavior control (nine questions), subjective norms (four questions), creativity (five questions), and entrepreneurial self-efficacy (22 questions). This study adopted previously tested and validated scales to measure the research variables. In order to ensure the accuracy of the language translation of each item in the scales, the scales were translated into Chinese and then a second translator back translated the scales to check for conceptual equivalency (Bhalla and Lin, 1987). For the measurement of entrepreneurial intention, the four questions were drawn from the scale of Liñán and Fayolle (2015). For the measurement of entrepreneurial attitude, the four questions were drawn from the scale of Obschonka et al. (2012). For the measurement of perceived behavior control, the nine questions were drawn from Prodan and Drnovsek’s (2010) scale. For the measurement of subjective norms, the four questions were drawn from Obschonka et al.’s (2012) scale. For the measurement of creativity, the five questions were drawn from Miranda et al. (2017). Lastly, for the measurement of entrepreneurial self-efficacy, the 22 questions drew on DeNoble et al.’s (1999) scale. All the above items were measured by Likert 5-point scales.

### Procedure

The survey was conducted in Zhejiang province, which has the most developed private economy and Internet economy in China. As one of the more developed provinces of China, Zhejiang province, also considered as China’s answer to Silicon Valley, is home to e-commerce giants like Alibaba Group Holding and NetEase. The technological start-ups in Zhejiang have contributed largely to its rising “new economy” where the provincial government has also capitalized on the role of entrepreneurship as its model for development (Zhang, 2018). The researchers first undertook a pilot study by distributing 100 questionnaires to students at Zhejiang University. Eighty two effective questionnaires were received, an effective questionnaire recovery rate of 82%. After testing and analysis, the CITC method was used to remove three questions with a correlation coefficient of less than 0.5. The final questionnaire was composed of 45 questions.

To reduce the sample selection bias, the survey was carried out in various locations with different cohorts and the questionnaires were administered randomly. To meet the research needs, from January 4, 2019 to February 28, 2019, the formal survey selected university students in Zhejiang as the sample selection range, controlled the respondents from the major, grade, gender and other aspects, and strived to meet the normal distribution of data. The coordinator contacted different universities in Zhejiang with an invitation to take part in the survey. If the universities agreed, they completed a registration form that indicated how many students would take part. The purpose of the research was outlined to the participants and a small incentive was provided to the respondents to compensate them for their time for completing the questionnaire. Six hundred and six questionnaires were distributed, and after excluding 83 invalid questionnaires, 523 valid questionnaires were obtained, a recovery rate of 86.45%.

## Results

### Exploratory Tests

In order to ensure the quality of the analysis results of the model, it is necessary to analyze the reliability and validity of the formal questionnaire data. In this study, statistical software SPSS20.0 was used to analyze the validity of six potential variables that are entrepreneurial self-efficacy, entrepreneurial attitude, subjective norms, perceived behavioral control, entrepreneurial intention and creativity.

From the reliability analysis of the questionnaire (Table 1), the Cronbach α coefficient of the six dimensions is between 0.859 and 0.931, which are all greater than 0.7, indicating that the six dimensions of the scale all have good internal consistency reliability.

**TABLE 1:** Reliability analysis results.

Item	Corrected item-total correlation	Cronbach’s alpha if item deleted	Cronbach’s alpha
Creativity 1	0.749	0.900	0.913
Creativity 2	0.813	0.886	
Creativity 3	0.769	0.896	
Creativity 4	0.791	0.891	
Creativity 5	0.773	0.895	
Subjective norm 1	0.681	0.833	0.859
Subjective norm 2	0.688	0.827	
Subjective norm 3	0.709	0.818	
Subjective norm 4	0.747	0.802	
Entrepreneurial attitude 1	0.757	0.846	0.883
Entrepreneurial attitude 2	0.784	0.835	
Entrepreneurial attitude 3	0.732	0.856	
Entrepreneurial attitude 4	0.717	0.862	
Perceived behavior control 1	0.712	0.916	0.924
Perceived behavior control 2	0.713	0.915	
Perceived behavior control 3	0.733	0.914	
Perceived behavior control 4	0.741	0.914	
Perceived behavior control 5	0.732	0.914	
Perceived behavior control 6	0.744	0.914	
Perceived behavior control 7	0.718	0.915	
Perceived behavior control 8	0.690	0.917	
Perceived behavior control 9	0.739	0.914	
Entrepreneurial intention 1	0.827	0.839	0.893
Entrepreneurial intention 2	0.789	0.854	
Entrepreneurial intention 3	0.777	0.858	
Entrepreneurial intention 4	0.670	0.887	
Entrepreneurial self-efficacy 1	0.773	0.922	0.931
Entrepreneurial self-efficacy 2	0.824	0.915	
Entrepreneurial self-efficacy 3	0.840	0.913	
Entrepreneurial self-efficacy 4	0.820	0.916	
Entrepreneurial self-efficacy 5	0.780	0.921	
Entrepreneurial self-efficacy 6	0.755	0.925	

From the validity analysis of the questionnaire (Table 2), it can be seen that the normalized load value of each measurement item in each dimension is greater than 0.7, the CR value of each dimension is greater than 0.7 and the AVE value is greater than 0.5, indicating that the scale has a good convergent validity.

**TABLE 2:** Convergent validity analysis results.

Latent variable	Item	Non-standard factor loading	Standard factor loading	Standard error	*T* value	CR	AVE
Creativity	Creativity 1	1.000	0.799			0.913	0.678
	Creativity 2	1.189	0.872	0.052	22.835***		
	Creativity 3	1.144	0.811	0.055	20.713***		
	Creativity 4	1.163	0.824	0.055	21.175***		
	Creativity 5	1.068	0.810	0.052	20.690***		
Subjective norms	Subjective norms 1	1.000	0.749			0.861	0.608
	Subjective norms 2	0.860	0.773	0.050	17.164***		
	Subjective norms 3	0.898	0.760	0.053	16.851***		
	Subjective norms 4	0.997	0.835	0.054	18.458***		
Entrepreneurial attitude	Entrepreneurial attitude 1	1.000	0.811			0.884	0.657
	Entrepreneurial attitude 2	1.026	0.861	0.046	22.435***		
	Entrepreneurial attitude 3	0.939	0.785	0.047	19.845***		
	Entrepreneurial attitude 4	0.836	0.783	0.042	19.792***		
Perceived behavior control	Perceived behavior control 1	1.000	0.741			0.574	0.928
	Perceived behavior control 2	1.001	0.746	0.058	17.281***		
	Perceived behavior control 3	1.052	0.755	0.060	17.516***		
	Perceived behavior control 4	1.051	0.774	0.058	18.005***		
	Perceived behavior control 5	0.977	0.752	0.056	17.426***		
	Perceived behavior control 6	0.994	0.782	0.055	18.202***		
	Perceived behavior control 7	1.012	0.758	0.058	17.593***		
	Perceived behavior control 8	1.027	0.725	0.061	16.763***		
	Perceived behavior control 9	1.066	0.783	0.058	18.227***		
Entrepreneurial intention	Entrepreneurial intention 1	1.000	0.888			0.897	0.685
	Entrepreneurial intention 2	0.930	0.845	0.036	25.607***		
	Entrepreneurial intention 4	1.000	0.854	0.038	26.129***		
	Entrepreneurial intention 3	0.824	0.714	0.043	19.361***		
Entrepreneurial self-efficacy	Entrepreneurial self-efficacy 1	1.000	0.829			0.932	0.696
	Entrepreneurial self-efficacy 2	1.049	0.857	0.043	24.199***		
	Entrepreneurial self-efficacy 3	1.038	0.869	0.042	24.762***		
	Entrepreneurial self-efficacy 4	1.060	0.854	0.044	24.104***		
	Entrepreneurial self-efficacy 5	0.954	0.812	0.043	22.257***		
	Entrepreneurial self-efficacy 6	0.991	0.781	0.047	21.024***		

***p < 0.001.

From the discriminant validity in Table 3 it can be seen that there is a positive correlation among the variables. The largest correlation coefficient between the studied constructs is 0.741 (between perceived behavior control and creativity). The figures also demonstrate that the square root of AVE value above the diagonal is greater than the correlative coefficient between the dimensions below the diagonal, indicating that the scale has a good discrimination validity.

**TABLE 3:** Discriminant validity analysis.

Latent variable	Creativity	Subjective norm	Entrepreneurial attitude	Perceived behavioral control	Entrepreneurial intention	Entrepreneurial self-efficacy
Creativity	**0.823**					
Subjective norms	0.569***	**0.780**				
Entrepreneurial attitude	0.653***	0.559***	**0.811**			
Perceived behavior control	0.676***	0.668***	0.741***	**0.758**		
Entrepreneurial intention	0.598***	0.440***	0.717***	0.682***	**0.828**	
Entrepreneurial self-efficacy	0.630***	0.601***	0.641***	0.727***	0.638***	**0.823**

The boldface above the diagonal is the square root of the AVE. ***p < 0.001.

According to Table 4, exploratory factor analysis of all the questions of the questionnaire shows that the KMO is equal to 0.960, greater than 0.7, and the significance of Bartlett’s test is less than 0.001, less than 0.05 indicating that the scale is suitable for factor analysis. In addition, the analysis results show that the cumulative variance explanation percentage of the first factor extracted is 35.434%, less than 40%, and also less than half of the total variance of 73.249%, indicating that there was no common method difference bias.

**TABLE 4:** KMO and Bartlett’s Test.

Kaiser–Meyer–Olkin measure of sampling adequacy		0.960
Bartlett’s test	Approx. chi-square	11615.558
	Df	496.000
	Sig.	<0.001

From Table 5’s exploratory factor analysis of the rotated component matrix, it can be seen that the maximum factor load of each question of the six dimensions is on the common factor, and the factor structure is coincided with the dimension structure of the questionnaire, indicating that the scale has a good result validity.

**TABLE 5:** Rotated component matrix.

Item	Factor 1	Factor 2	Factor 3	Factor 4	Factor 5	Factor 6
Perceived behavior control 5	0.723					
Perceived behavior control 3	0.718					
Perceived behavior control 1	0.669					
Perceived behavior control 4	0.662					
Perceived behavior control 2	0.646					
Perceived behavior control 6	0.642					
Perceived behavior control 8	0.601					
Perceived behavior control 7	0.591					
Perceived behavior control 9	0.590					
Entrepreneurial self-efficacy 3		0.798				
Entrepreneurial self-efficacy 2		0.786				
Entrepreneurial self-efficacy 6		0.735				
Entrepreneurial self-efficacy 4		0.732				
Entrepreneurial self-efficacy 5		0.719				
Entrepreneurial self-efficacy 1		0.578				
Entrepreneurial intention 1			0.764			
Entrepreneurial intention 2			0.739			
Entrepreneurial intention 4			0.733			
Entrepreneurial intention 3			0.709			
Subjective norms 3				0.822		
Subjective norms 4				0.764		
Subjective norms 1				0.730		
Subjective norms 2				0.703		
Entrepreneurial attitude 1					0.746	
Entrepreneurial attitude 2					0.688	
Entrepreneurial attitude 3					0.684	
Entrepreneurial attitude 4					0.615	
Creativity 5						0.689
Creativity 4						0.677
Creativity 2						0.606
Creativity 3						0.594
Creativity 1						0.570

*Varimax is used.*

### Model Analysis

The model is analyzed by hierarchical regression, and the test results of Model 1 and Model 2 in Table 6 illustrated that entrepreneurial self-efficacy has a significant positive effect on perceived behavior control (β = 0.727, *p* < 0.001). According to the test results of Model 3 and Model 4, entrepreneurial attitude, subjective norm, perceived behavior control and creativity all have a significant positive influence on entrepreneurial intention (β1 = 0.387, *p*1 < 0.001; β2 = 0.234, *p*2 < 0.001; β3 = 0.103, *p*3 = 0.007; β4 = 0.136, *p*4 = 0.001).

**TABLE 6:** Results of regression analysis.

Variable types	Perceived behavior control		Entrepreneurial intention		Entrepreneurial intention					
	Model 1	Model 2	Model 3	Model 4	Model 5	Model 6	Model 7	Model 8	Model 9	Model 10
**Control variable**										
Gender	−0.044 (0.062)	0.013 (0.043)	−0.140** (0.074)	−0.086** (0.052)	−0.140** (0.074)	−0.130*** (0.054)	−0.140** (0.074)	−0.055 (0.063)	−0.140** (0.074)	−0.077 (0.056)
Sophomore (compared with freshman)	0.082 (0.072)	0.051 (0.050)	0.004 (0.086)	−0.039 (0.060)	0.004 (0.086)	−0.010 (0.062)	0.004 (0.086)	−0.025 (0.071)	0.004 (0.086)	−0.038 (0.064)
Junior (compared with freshman)	0.021 (0.094)	0.015 (0.065)	−0.018 (0.112)	−0.007 (0.078)	−0.018 (0.112)	−0.036 (0.081)	−0.018 (0.112)	0.019 (0.093)	−0.018 (0.112)	−0.019 (0.084)
Senior (compared with freshman)	0.001 (0.117)	0.025 (0.080)	−0.010 (0.139)	−0.041 (0.097)	−0.010 (0.139)	−0.028 (0.100)	−0.010 (0.139)	−0.057 (0.115)	−0.010 (0.139)	−0.024 (0.104)
Family entrepreneurship	−0.153*** (0.061)	−0.047 (0.043)	−0.056 (0.073)	0.062* (0.052)	−0.056 (0.073)	0.022 (0.053)	−0.056 (0.073)	0.057 (0.062)	−0.056 (0.073)	0.065* (0.056)
**Independent variable**										
Entrepreneurial self-efficacy		0.725*** (0.034)								
Entrepreneurial attitude				0.387*** (0.043)		0.348** (0.114)				
Subjective norms				0.234*** (0.043)				0.311** (0.129)		
Perceived behavior control				0.103** (0.056)						0.161 (0.133)
**Moderator variable**										
Creativity				0.136** (0.049)		0.497** (0.12)		0.056 (0.15)		−0.090 (0.146)
**Interaction**										
Creativity × entrepreneurial attitude						−0.136 (0.03)				
Creativity × subjective norms								0.372* (0.04)		
Creativity × perceived behavior control										0.608*** (0.041)
*R*^2^	0.033	0.543	0.022	0.535	0.022	0.377	0.022	0.498	0.022	0.459
Δ*R*^2^	0.033	0.510	0.022	0.513	0.022	0.355	0.022	0.476	0.022	0.438
*F*-value	3.513***	102.014***	2.313*	65.613***	2.313*	38.861***	2.313*	63.714	2.313*	54.598***

*p < 0.05; **p < 0.01; ***p < 0.001.

**FIGURE 2:**
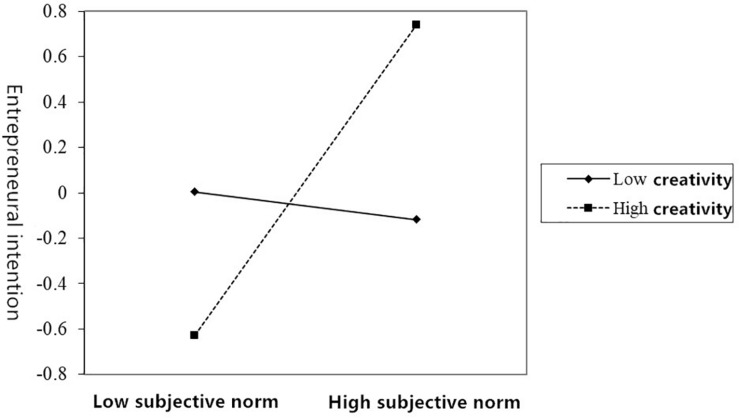
A line chart of the moderating effect of creativity between subjective norms and entrepreneurial intention.

**FIGURE 3:**
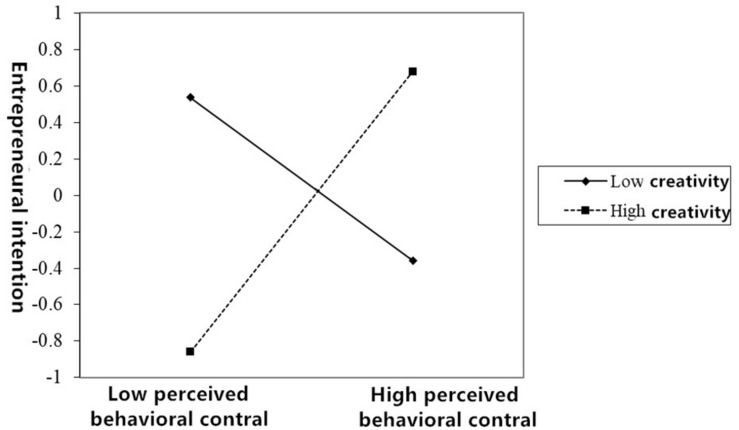
A line chart of the moderating effect of creativity between perceived behavior control and entrepreneurial intention.

Model 5 and Model 6 indicate the moderating effect of creativity between entrepreneurial attitude and entrepreneurial intention. From the results of the analysis, it can be seen that the interaction between creativity and entrepreneurial attitude is not significant (β = −0.136, *p* > 0.05), indicating that creativity has no moderating effect between entrepreneurial attitude and entrepreneurial intention. Model 7 and Model 8 verify the moderating effect of creativity between subjective norms and entrepreneurial intentions. From the analysis results and Figure 2, the interaction between creativity and subjective norms is significantly positive (β = 0.372, *p* < 0.001), indicating that creativity plays a significant and positive role in moderating subjective norms and entrepreneurial intentions. Model 9 and Model 10 verify the moderating effect of creativity between perceived behavior control and entrepreneurial intention. From the analysis results and Figure 3, it can be seen that the interaction between creativity and perceived behavior control is significantly positive (β = 0.608, *p* < 0.001), indicating that creativity plays a significant and positive role in moderating the action of perceived behavior control on entrepreneurial intention.

## Discussion and Conclusion

This paper uses SPSS20.0 and Amos 22.0 software to construct the SEM structural equation model, and analyzes the influence of creativity on entrepreneurial intention, based on 523 valid questionnaires from college students. The results show that creativity has a positive influence on entrepreneurial intention, but it is the entrepreneurial attitude that is the most important factor that affects entrepreneurial intention rather than creativity, followed by the perceived behavioral control, and then the subjective norms. In addition, creativity has a positive moderating effect on both the actions of perceived behavioral control and subjective norms on entrepreneurial intention. The results also show that entrepreneurial self-efficacy has a positive influence on the perception of behavioral control. To sum up the results hypotheses, H1, H2, H3, H4, H4b, H4c, and H5 were statistically proven, whilst H4a was not statistically supported. The results of this research support Ajzen’s (1991) TPB model and the three attitudes that lead to intent. The factor that is considered to be the weakest attitude, perceived social norms (Ajzen, 1991; Liñán et al., 2011) was found to be significant in this research, which supports the contention that it is context specific and is a significant factor in China. This type of influence can be more significant in some contexts (Fayolle and Gailly, 2015). Since entrepreneurial attitude is the strongest factor influencing entrepreneurial intention, any mediation effect through creativity may be relatively insignificant in comparison. Finally, the importance of personal entrepreneurial attitude and perceived behavioral control to entrepreneurial intent in this research supports previous findings (e.g., Liñán et al., 2011). This paper has the following theoretical significance: Firstly, the need to broaden the scope of creativity research. In the past, the field of creativity research was mainly focused in economic organizations, like enterprises and research and design institutions, and the research subjects were mostly enterprise leaders or research and design personnel. In this study, the theory of creativity has been applied to the field of higher education, and college students were taken as the research subjects to broaden the scope of creativity research. Secondly, the need to expand research into the influencing factors of entrepreneurial intention. Although previous studies have also paid close attention to the influence of individual factors on entrepreneurial intention, creativity is still an easily neglected individual factor. This study introduces creativity as an individual factor to extend the influencing factors of entrepreneurial intention. This also confirms the research conclusions and viewpoints of Ahlin et al. (2014) and Khedhaouria et al. (2015), that is, creativity can be regarded as a valuable ‘raw material’ owned by individuals, which can promote the enhancement of entrepreneurial intention by improving the awareness and skills related to entrepreneurship, such as opportunity identification, etc.

Based on the above analysis, this study believes that in the context of China’s current economic transformation and upgrading, to achieve the upgraded version of “mass entrepreneurship and innovation” for college students, efforts can be made from the following aspects. First, colleges and universities could continue to improve the quality of their entrepreneurship education. This can be done by expanding the teaching material for entrepreneurship modules to cultivate students’ creativity and by improving teaching methods for entrepreneurship education to improve students’ understanding of entrepreneurship and stimulate students’ creativity. This can include a range of different learning experiences, including not only in-class teaching, but also extra-curricular activities, which have been found to be particularly efficacious in developing students’ entrepreneurial mindsets in the Chinese context (Cui et al., 2019). This research has highlighted the role that Ajzen’s (1991) three TPB attitudes, and creativity, play in entrepreneurial intent. Based on this research, to develop entrepreneurial intent, both individually and more widely, entrepreneurship education should focus on positively developing the three attitudes, and individuals’ creativity. Previous research has found that entrepreneurship education can efficiently develop creativity, which can successfully nurture entrepreneurial intentions (Shahab et al., 2019). Active experiential approaches can be beneficial in entrepreneurship education (Fuchs et al., 2008), particularly those that provide an authentic learning experience. Authentic learning experiences can encourage deeper learning, encourage engagement and more effectively prepare students (Macht and Ball, 2016). For example, hands-on entrepreneurial experiences can help to develop intent and stimulate creativity, which can be further developed by the creation of specific goals and the sense of competition (Bell and Bell, 2016).

Secondly, the government could create a better entrepreneurial environment for university entrepreneurs. Preferential policies could be provided for college students, such as setting up a special entrepreneurship support fund, providing free business premises, and providing tutorials. Furthermore, establishing an effective entrepreneurial atmosphere across whole society would encourage young people to innovate and start their own businesses. This could be achieved by holding entrepreneurship competitions, media publicity, and other forms of promotion. Finally, the increased joint ventures between universities and industries, financial institutions and/or relevant associations would create an entrepreneurial ecosystem and encourage innovation. Developing and tailoring entrepreneurial ecosystems conducive to the support of entrepreneurship education has been highlighted as an effective way to support the development of students’ entrepreneurial attitudes and abilities (Bell, 2019).

## Limitations

Although this research expands the use of the TPB model to study the relationship between creativity and entrepreneurial intention, it also has practical significance for entrepreneurship education. However, in common with all research, some potential limitations exist and will now be considered along with future research opportunities. Firstly, this paper uses self-assessment to measure individual creativity, which may deviate from the actual situation. In future research, we can use mutual assessment or test, to obtain a deeper and more objective measurement of individual creativity. Secondly, this research focuses on the impact of creativity on individual entrepreneurial intention. Future research can explore the impact of entrepreneurs’ creativity in entrepreneurial teams, entrepreneurial performance and entrepreneurial growth, which can further expand the research area. Finally, research can be undertaken to investigate across a range of universities, and the number of samples can be increased to improve the external validity of the research conclusions.

## Data Availability Statement

The original contributions presented in the study are included in the article/supplementary material, further inquiries can be directed to the corresponding author/s.

## Ethics Statement

The study was reviewed and approved by Wenzhou University Ethics Committee. Written informed consent to participate in this study was provided by the participants.

## Author Contributions

YS designed the work. YS and JW designed the field survey. TY assisted in the data collection. YS, TY, RB, and JW analyzed the data and experiment result. YS, RB, and WJ wrote the manuscript.

## Conflict of Interest

The authors declare that the research was conducted in the absence of any commercial or financial relationships that could be construed as a potential conflict of interest.
